# *Chryseobacterium arthrosphaerae* ventriculitis: A case report

**DOI:** 10.1097/MD.0000000000021751

**Published:** 2020-08-21

**Authors:** Jae Hyoung Im, Donghwi Kim, Jin Ju Kim, Eun Young Kim, Young Kyoung Park, Hea Yoon Kwon, Moon-Hyun Chung, Ji Hyeon Baek, Jin-Soo Lee

**Affiliations:** a Division of Infectious diseases, Department of Internal Medicine; b Department of Internal Medicine; c Department of Laboratory Medicine; d Department of Neurosurgery; e Translation Research Center, Inha University College of Medicine, Incheon; f Department of Internal Medicine, Seogwipo Medical Center, Jeju, Republic of Korea.

**Keywords:** catheter-Related Infections, catheters, central nervous system infections, *Chryseobacterium*, indwelling

## Abstract

**Introduction:**

Chryseobacterium arthrosphaerae is a gram-negative bacteria, known for its intrinsic multidrug resistance, which can lead to treatment difficulties.

**Patient concerns:**

A 56-year-old male had an indwelling external ventricular drainage catheter for 6 months and had been frequently treated with antibiotics for nosocomial infections. He showed cerebrospinal fluid pleocytosis and an abrupt fever during hospitalization.

**Diagnosis:**

He was diagnosed as a ventriculitis caused by *Chryseobacterium arthrosphaerae (C arthrosphaerae)*.

**Intervention:**

Initially, we used ciprofloxacin as the backbone in combination with minocycline (and rifampin). However, fever and pleocytosis persisted, and improvement was slow. We then switched the minocycline and rifampin regiment to trimethoprim/sulfamethoxazole. Following this switch of antibiotics, the patient's pleocytosis rapidly improved, allowing the replacement of his external ventricular drainage catheters. *C arthrospharae* was no longer growing in cerebrospinal fluid and he was recovered from ventriculitis.

**Outcomes:**

The patient remains alive without any incidence of *C arthrosphaerae* recurrence.

**Conclusion:**

We propose trimethoprim/sulfamethoxazole alone or in combination with ciprofloxacin to be good candidates for the treatment of ventriculitis by *C arthrosphaerae*.

## 1 Introduction

The genus *Chryseobacterium* comprises of aerobic and non-fermentative gram-negative bacilli belonging to the Flavobacteriaceae family.^[1]^ In 1984, *Flavobacterium gleum* was first reported in humans^[2]^ and was subsequently re-classified as a *Chryseobacterium* species in 1994.^[3]^ Most *Chryseobacterium* spp. are isolated from the environment such as soil and water; however, *C indologenes* and *C gleum* have been found in catheter-related infections, ventilator-associated pneumonia and other infections in humans.^[4]^*C arthrosphaerae* was first isolated from the excrement of *Arthrosphaera magna Attems* in India in 2010.^[5]^ Although *C arthrosphaereae* has rarely been reported to cause infection in humans,^[6]^ we have experienced cases of central nervous system (CNS) infection by multidrug-resistant *C arthrosphaereae*. Here, we report the clinical course of a patient with CNS infection by *C arthrosphaereae* and his successful treatment using trimethoprim/sulfamethoxazole and ciprofloxacin.

## 2 Case presentation

A 56-year-old man with diabetics, chronic kidney disease, hypertension, and alcoholic liver cirrhosis was admitted to Inha university hospital for a brain abscess with ventriculitis. Prior to this event, he had been treated with external ventricular drainage catheters and a ventriculoperitoneal shunt for hydrocephalus as sequelae of the ventriculitis. Upon shunt infection, his ventriculoperitoneal shunt had been removed, and he had been treated with 2 indwelling external ventricular drainage catheters for 6 months. He had been frequently treated with antibiotics for recurrent urinary tract infections and pneumonia during hospitalization. On the 560th day of hospitalization, he developed an abrupt fever (38°C). On laboratory examination, he showed elevated C-reactive protein (4.41 mg/dL) and cerebrospinal fluid (CSF) pleocytosis. In his CSF, white blood cell (WBC) count was 450/mm^3^, lactic acid was 5.95 mg/dL, protein was 109.5 mg/dL and glucose was 52 mg/dL (Table 1). He was diagnosed with a catheter-related CNS infection. Considering his history of frequent antibiotics use, we started him on meropenem (1 g every 12 hours). On the third day of the event, *Chryseobacterium* spp. was isolated from CSF by BACTEC^TM^. The bacterial DNA was extracted and 16S rRNA gene sequence was analyzed for species identification. Polymerase chain reaction was performed using the universal primers 27F and 1492R. The polymerase chain reaction product was sequenced at Macrogen Inc. (Seoul, Korea). The nucleotide sequence was aligned by GeneBank BLAST (Available at: https://blast.ncbi.nlm.nih.gov/Blast.cgi) and TrueBac ID (Available at: https://www.truebacid.com/). The sequence showed 99.93% similarity with *Crysoebacterium arthrosphaerae* by both alignment tools. The Minimum Inhibitory Concentration (MIC) for *C arthrosphaerae* are shown in Table 2. We used ciprofloxacin (500 mg every 12 hours) as a backbone and added minocycline (200 mg loading and 100 mg every 12 hours). We initially added minocycline rather than trimethoprim/sulfamethoxazole because of his lower estimated glomerular filtration rate (24 mL/min/1.73m^2^) and previous history of chronic kidney disease exacerbation. However, the patient's clinical improvement was not significant. We, therefore, added rifampin (600 mg every 24 hours) on the 14th day of the event. Following this change, the patient's fever improved, but his left ventricle CSF still presented pleocytosis (WBC: 450/uL). On the 21st day of the event, we switched to a combination of ciprofloxacin and trimethoprim/sulfamethoxazole (5 mg/kg every 12 hours), with ciprofloxacin given as intraventricular therapy. Although ciprofloxacin intraventricular therapy was discontinued due to seizure, his CSF pleocytosis improved (to WBC 5/mm^3^) with the ciprofloxacin and trimethoprim/sulfamethoxazole combination. On the 35th day of the event, we replaced his external ventricular drainage catheters, and maintained ciprofloxacin for 5 weeks. Along with the ciprofloxacin, high-dose sulbactam was used for a post-operative *Acinetobacter baumanii* infection. After 5 weeks, all antibiotics were discontinued. The patient remains alive without any incidence of *C arthrosphaerae* recurrence.

**Table 1:**
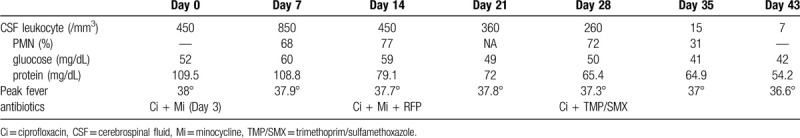
Changes in CSF and body temperature as antibiotics are used.

**Table 2:**
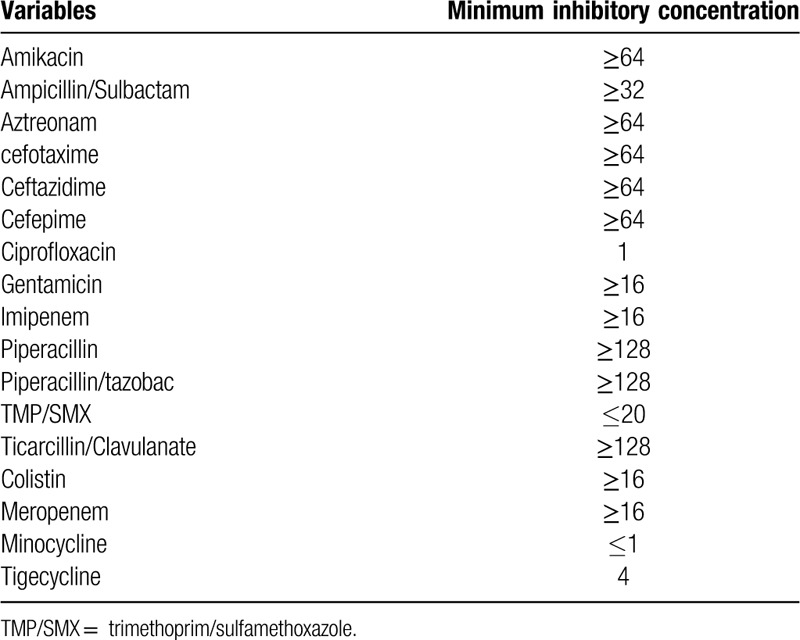
Intial antibiotics susceptibility of *Chryseobacterium arthrosphaerae*.

## 3 Discussion

*Chryseobacterium* spp. are well known for their intrinsic resistance to a wide spectrum of antibiotics, such as cephalosporin, carbapenems, aminoglycoside, linezolid, and polymixin. As a substitute, they may be sensitive to fluoroquinolones, macrolides, and rifampin. The clinical data on *C arthrosphaerae* are very limited. A previous study reported *C arthrosphaerae* with resistance to all antibiotics.^[7]^ Moreover, no standard guidelines are available for the antimicrobial susceptibility of the *Chryseobacterium* genus. For MIC interpretation, some clinicians have used breakpoint for *Staphlyococcus aureus*, while others have selected cutoffs for nonfermenting gram-negative bacilli.^[8–10]^

We encountered several difficulties in the treatment of this case, including

(1) the multidrug resistance of *Chryseobacterium*,
(2) an unexpected clinical finding of *C arthrosphaerae*, and
(3) choice of antibiotics due to the patient's chronic kidney disease.

*Chryseobacterium spp.* are resistant to cephalosporin and carbapenem, which are commonly used for CNS infection. In this case, trimethoprim/sulfamethoxazole showed good clinical effectiveness. Although reports on *C arthrosphaerae* are extremely rare, it is noteworthy that trimethoprim/sulfamethoxazole was successful in the treatment of meningitis caused by *Elizabethkingia meningosepticum,* which was previously classified as a member of the *Chryseobacterium* genus.^[11]^ In addition, trimethoprim/sulfamethoxazole can also be used to treat other hospital-acquired meningitis cases, such as *Staphylococcus* and *Enterobacteriacae*.^[12]^ Trimethoprim/sulfamethoxazole can be considered a good candidate for CNS treatment of *C arthrosphaerae*.

The *C arthrosphaerae* isolated from the patient showed a MIC of 1 to 2 for ciprofloxacin. We could not predict the therapeutic effect of ciprofloxacin. The patient showed clinical improvement after combination therapy, but the specific effect of the combination has not been clearly defined, and no studies have investigated synergy or antagonism in this context. Considering the relative scarcity of available drugs for *Chryseobacterium*, further studies on combination therapy are needed. It should also be noted that the MIC increased from 1-2 to 2–4 during the use of ciprofloxacin in this patient. The use of antibiotics carries the potential for both inducible and heterogenous resistance. However, the resistance mechanism of *C arthrosphaerae* remains poorly understood.^[7]^ Further study of *C arthrosphaerae* antibiotic resistance is needed.

As in this case, the treatment of *Chryseobacterium spp*. is difficult, so efforts to reduce the infection are important. Long-term admission, invasive intervention, invasive catheters, and a wide range of antibiotics have been reported as risk factors for *Chryseobacterium* infection. Our patient had long-term hospital admission, including intensive care unit admission, long-term indwelling catheters, and was frequently exposed to broad-spectrum antibiotics. It is important to reduce these risk factors when considering the emergence of various multidrug-resistant bacteria.

In conclusion, we have experienced CNS infection by *C arthrosphaerae* accompanied by broad antibiotic resistance. We successfully treated patient with combinatorial antibiotics therapy (ciprofloxacin and trimethoprim/sulfamethoxazole). However, further research is necessary to understand the resistance mechanism of *C arthrosphaerae,* which in turn can help make better treatment regiments in the future.

## Author contributions

Conceptualization: Jae Hyoung Im and Jin-Soo Lee. Writing manuscript: Jae Hyoung Im. Identification of pathogen: Jin Ju Kim and Young Kyoung Park. Case discussion and interpretation: Donghwi Kim, Eun Young Kim, Hea Yoon Kwon, Moon-Hyun Chung, and Ji Hyeon Baek. Revision of manuscript: Jin-soo Lee. Final approval of manuscript: All authors.
